# Acute Enlargement of a Frontal Calvarial Lesion in Polyostotic Fibrous Dysplasia Associated with McCune–Albright Syndrome: A Case Report

**DOI:** 10.1055/a-2947-3017

**Published:** 2026-10-15

**Authors:** David Zhang, Rachel A. Multz, Pouya Jamshidi, Alison M. Boyce, Allison J. Hahr, Stephen T. Magill

**Affiliations:** 1 Department of Neurological Surgery 12244 Northwestern University Feinberg School of MedicineChicagoIllinoisUnited States; 2 Department of Pathology 12244 Northwestern University Feinberg School of MedicineChicagoIllinoisUnited States; 3 2511 Metabolic Bone Disorders Unit, National Institute of Dental and Craniofacial Research, National Institutes of HealthBethesdaMarylandUnited States; 4 Division of Endocrinology, Metabolism and Molecular Medicine 12244 Northwestern University Feinberg School of MedicineChicagoIllinoisUnited States

**Keywords:** McCune–Albright, aneurysmal bone cyst, fibrous dysplasia, bone lesion

## Abstract

**Background** McCune–Albright syndrome is a rare mosaic disorder caused by postzygotic activating mutations in *GNAS* , classically presenting with fibrous dysplasia (FD), hyperpigmented macules, and endocrinopathies. Craniofacial FD is common and typically follows a benign course. However, acute symptomatic changes with aggressive imaging features may mimic malignant transformation or secondary lesions such as aneurysmal bone cysts (ABCs), creating significant diagnostic uncertainty.

**Case Description** We report a 20-year-old woman with known MAS and polyostotic FD who presented with a 4-week history of progressive left frontal swelling, headache, and tenderness. Interval imaging revealed a rapidly enlarging erosive lytic lesion of the left frontal calvarium with peripheral enhancement and an internal fluid–fluid level, raising concern for secondary pathology or malignancy. Given rapid growth and cortical erosion, the patient underwent surgical resection with cranioplasty. Intraoperatively, the lesion contained serosanguinous fluid. Histopathology demonstrated bland spindle cell proliferation with curvilinear woven bone trabeculae and hemosiderin-laden macrophages, without cytologic atypia, necrosis, or features of ABC. Molecular testing confirmed a *GNAS* p.(R201C) mutation, consistent with FD. The patient had an uncomplicated postoperative course with complete symptom resolution.

**Conclusion** This case highlights that fluid–fluid levels, rapid interval growth, and erosive changes can occur in craniofacial FD without ABC formation or malignant transformation. Imaging findings alone are insufficient to distinguish benign changes from aggressive pathology. Accurate diagnosis requires a multimodal approach integrating clinical context, advanced imaging, histopathology, and pathologic analysis. Surgical intervention is justified in some cases to establish diagnosis and provide symptomatic relief.

## Introduction

McCune–Albright syndrome is a rare mosaic disorder characterized by the triad of fibrous dysplasia (FD), hyperpigmented skin macules, and hyperfunctioning endocrinopathies, arising from somatic activating mutations in the *GNAS* gene. 1 2 The GNAS mutation leads to constitutive activation of the Gαs protein and inappropriate production of intracellular cyclic adenosine monophosphate (AMP), resulting in impaired differentiation of skeletal stem cells and replacement of normal bone with expansile fibro-osseous tissue. 1 The craniofacial skeleton is one of the most commonly affected sites in FD, occurring in approximately 50% of patients with documented polyostotic FD. 3

While FD lesions typically follow a gradual, asymptomatic course with progressive expansion throughout childhood and early adolescence, stabilizing after puberty, certain presentations can produce acute clinical deterioration that mimics aggressive pathology. 4 5 Aneurysmal bone cysts (ABCs) are rapidly expanding fluid-filled lesions that can form within preexisting areas of FD, manifesting with acute onset of severe pain, rapidly expanding localized deformity, and potentially vision loss when compressing critical structures. 6 These secondary lesions occur in a small fraction of skull base FD cases and present a diagnostic challenge, as their aggressive radiographic appearance, characterized by cortical destruction, peripheral enhancement, and internal fluid–fluid levels, can mimic malignant transformation. 7 8 Malignant transformation occurs in approximately 1–2% of FD cases, most commonly as osteosarcoma and less frequently as fibrosarcoma or chondrosarcoma. 9

Distinguishing secondary ABC transformation, hemorrhagic transformation, and other benign changes from true malignant transformation is critical, as malignancy is rare but carries significant morbidity and mortality. 10 Imaging features alone, however, make it difficult to differentiate between benign and malignant transformation, as fluid–fluid levels and erosive changes may occur in both cases. 11 The presence of an erosive lytic lesion with rapid evolution in a patient with known FD necessitates a comprehensive evaluation through analysis of clinical presentation, advanced imaging, histopathology, and molecular testing. 8 12 13

This case highlights the diagnostic complexity of acute presentations in craniofacial FD and underscores the importance of multimodal diagnostic assessment in distinguishing benign complications from malignant degeneration, particularly when imaging findings suggest aggressive behavior.

## Case Presentation

### History

A 20-year-old woman with a history of McCune–Albright syndrome and polyostotic FD presented with a 3- to 4-week history of left frontal swelling associated with local tenderness and headache, which had spontaneously resolved by the time of evaluation. Six months prior, the patient had initiated a hormonal contraceptive patch, which was discontinued 1 month prior to presentation; her menstrual cycle had not yet returned to baseline. She reported a 5-pound unintentional weight loss but denied fevers, fatigue, night sweats, or other systemic symptoms. Her vision had remained stable for several years. Neurological examination showed normal speech, intact extraocular movements without diplopia, symmetric facial function, full strength, intact sensation, and a normal gait.

Review of head computed tomography (CT) imaging from 5 years prior revealed stable FD involving the left skull base, preorbital, nasal, and lateral temporal regions. However, interval CT imaging demonstrated a new erosive lytic lesion in the left frontal calvarium with peripheral enhancement and an internal fluid–fluid level, raising concern for rapid structural change or secondary pathology. Over the span of 6 weeks, the lesion enlarged from approximately 12 to 19 mm in maximal diameter at the outer table, with progressive thinning and near-complete erosion of the frontal calvarium ( Fig. 1 ).

**Fig. 1:**
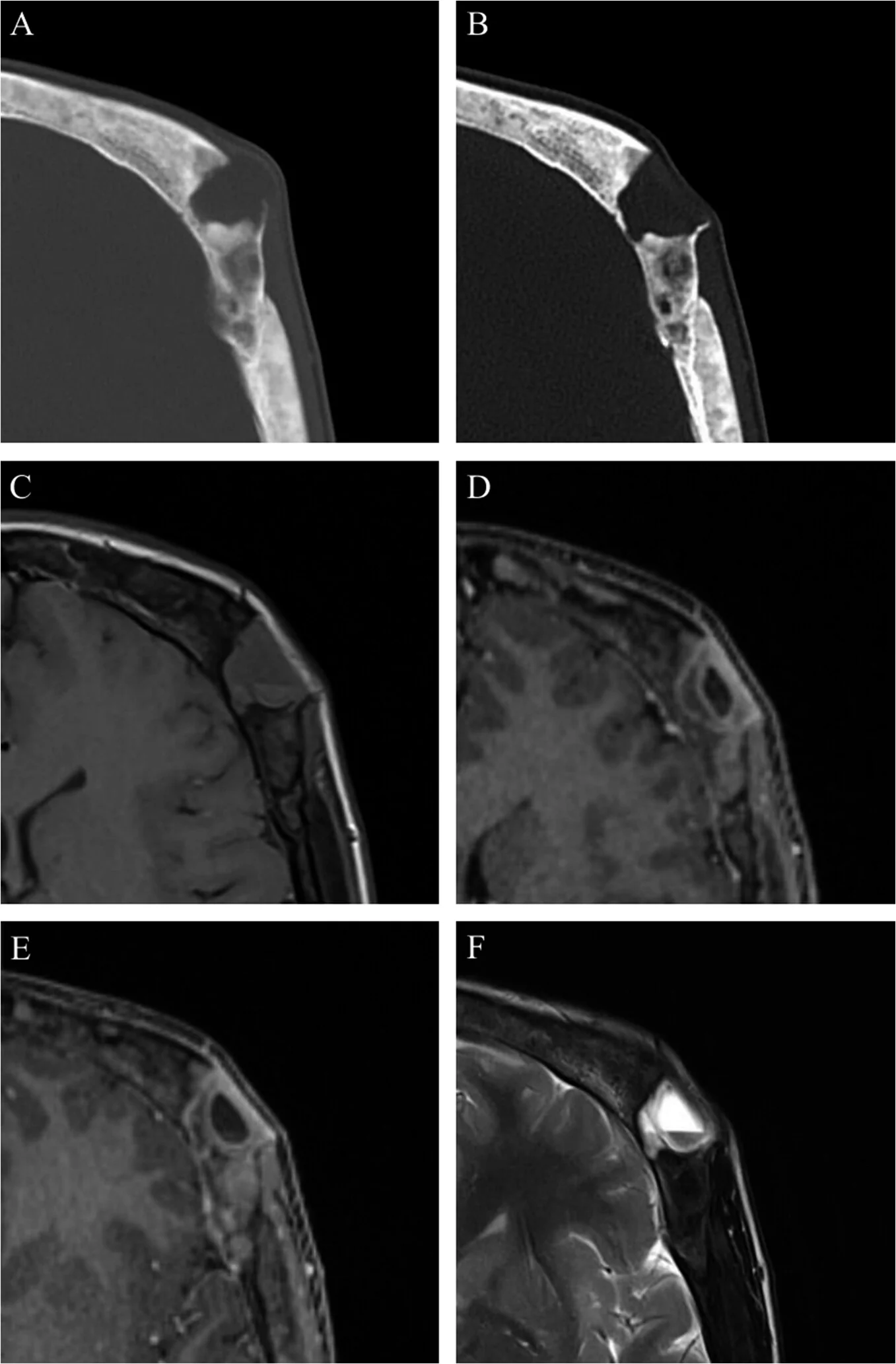
Computed tomography (CT) head without contrast at presentation ( **A** ), CT head without contrast 6 weeks after presentation ( **B** ), T1-weighted magnetic resonance imaging (MRI) brain precontrast 6 weeks after presentation ( **C** ), T1-weighted MRI brain postcontrast 6 weeks after presentation ( **D, E** ), T2-weighted MRI brain postcontrast 6 weeks after presentation ( **F** ).

### Procedure

Given the lesion’s rapid interval growth and concern for malignant transformation or development of an ABC, the patient underwent left frontal resection with custom hydroxyapatite cranioplasty for diagnostic and therapeutic purposes. Intraoperatively, the lesion contained serosanguinous fluid consistent with the fluid–fluid level seen on imaging. The surrounding bony margins were remodeled, and the calvarial defect was reconstructed using hydroxyapatite bone cement to achieve an optimal cosmetic outcome. The procedure was completed without intraoperative complication.

The lytic lesion tissue was analyzed with the Expanded Solid Tumor next generation sequencing (NGS) panel and the FusionPlex Solid Tumor NGS panel. Postoperative molecular testing confirmed a GNAS p.(R201C) alteration, consistent with the known molecular etiology of FD. 14 Histopathology demonstrated a spindle cell proliferation embedded in variably collagenous stroma with fragments of curvilinear woven bone trabeculae and hemosiderin-laden macrophages ( Fig. 2 ). The spindle cells were cytologically bland, with ramiform amphophilic cytoplasm, angulated nuclei, and evenly staining basophilic chromatin ( Fig. 2 ). The vasculature consisted of small, thin-walled vessels and capillaries, and scattered mast cells were present ( Fig. 2 ). Mitotic activity was inconspicuous, and no cytologic atypia or necrosis was identified. Importantly, there were no features suggestive of ABC, such as cavernous blood-filled spaces or septations, and no histologic evidence of malignancy or sarcomatous transformation. 6

**Fig. 2:**
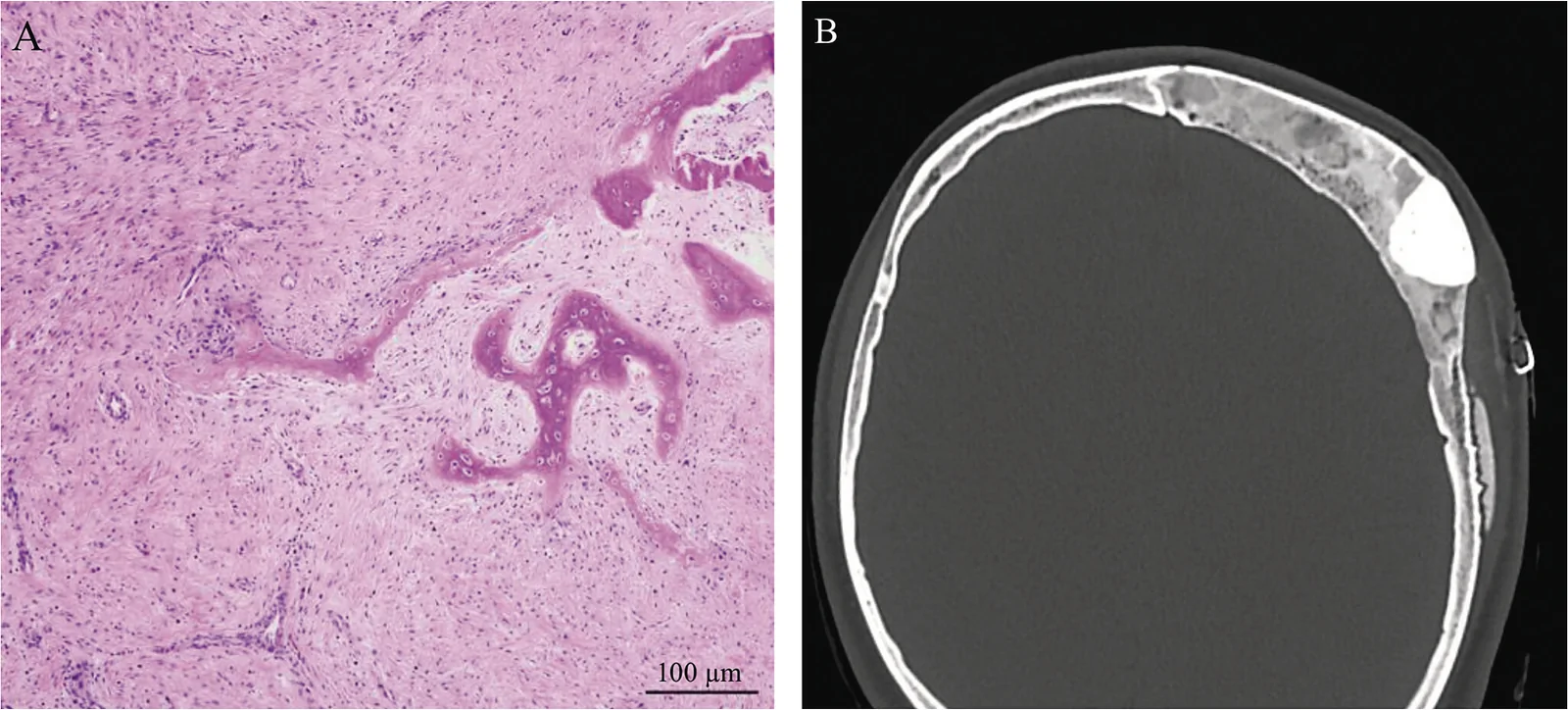
Hematoxylin and eosin-stained sections demonstrate a spindle cell proliferation embedded in variably collagenous stroma with fragments of curvilinear trabeculae of woven bone ( **A** ). Immediate postoperative axial computed tomography head without contrast shows hydroxyapatite in situ cranioplasty ( **B** ).

### Postoperative Course

The patient tolerated the procedure well, and postoperatively she recovered in post-anesthesia care unit and then transferred to the neurosurgical floor in stable condition. The patient was discharged on postoperative day 1.

At 2-week postoperative follow-up, the patient remained neurologically intact with excellent wound healing and no recurrence of swelling, erythema, pain, or headache, and excellent cosmesis. She had no recurrence at 3 months.

## Discussion

### Fluid–Fluid Levels without Aneurysmal Bone Cyst

The presence of fluid–fluid levels on imaging is often associated with ABCs, which occur in a small fraction of craniofacial FD cases and represent a common cause of acute clinical deterioration. 8 15 16 However, in this case, the serosanguinous fluid identified intraoperatively and hemosiderin-laden macrophages on histopathology, combined with the absence of cavernous blood-filled spaces or septations characteristic of ABC, suggest that fluid accumulation can occur within FD lesions themselves without meeting full histologic criteria for secondary ABC formation. 12 15 17 18

The mechanism underlying this acute presentation remains uncertain. Possibilities include transient vascular leak within the highly vascular FD tissue or other inflammatory processes. 5 19 Periorbital inflammation associated with vascular leak has been described in three patients with craniofacial FD presenting with recurrent episodes of pain, edema, and erythema, all of whom had cystic changes on imaging. 19 In those patients, this inflammatory process was effectively managed with corticosteroids and may explain transient symptomatic presentations that resolve spontaneously. 19 In contrast, our patient’s symptoms had already resolved at evaluation, and the primary concern was a rapidly enlarging lesion; therefore, corticosteroids would not have addressed the diagnostic uncertainty and could have masked malignancy. Hormonal changes may also be relevant. FD expresses estrogen receptors, and estrogen exposure has been linked to lesion progression, so our patient’s recent contraceptive exposure may have contributed, although causation cannot be established. 20 21 For definitive diagnosis and subsequent cranioplasty, we opted for open resection rather than needle aspiration, which is often non-diagnostic in FD and would not have allowed reconstruction of the skull defect. 22

### Imaging Heterogeneity in Fibrous Dysplasia

Craniofacial FD exhibits highly variable signal intensities on magnetic resonance imaging (MRI), with T2-weighted images showing heterogeneous patterns and contrast enhancement in many cases. 23 24 25 26 This heterogeneity reflects underlying biological variability in lesion activity and composition, and imaging features alone cannot reliably differentiate benign changes from malignant transformation. 23 24 25 26 Both benign and aggressive lesions may demonstrate cortical destruction, peripheral enhancement, and fluid–fluid levels, creating diagnostic uncertainty. 11 24

### Distinguishing Benign from Malignant Transformation

Malignant transformation of craniofacial FD is rare but represents a critical diagnostic consideration given its significant morbidity. 17 27 28 Locally aggressive FD, an extremely rare variant, can present with “cortical destruction and extension into soft tissue” that radiographically suggests malignancy yet proves benign on histopathology. 29 In this case, the peripheral enhancement and erosive lytic changes appropriately raised concern for malignant transformation, justifying surgical intervention for proper diagnosis. 29

The molecular confirmation of GNAS p.(R201C) mutation provided critical diagnostic certainty consistent with the known molecular etiology of FD. 1 However, the *GNAS* mutation alone cannot distinguish benign from malignant lesions. 5 The absence of cytologic atypia, necrosis, and malignant histologic features on pathology, combined with the *GNAS* mutation and clinical context, confirmed the diagnosis of FD without transformation. 13

### Importance of Multimodal Diagnostic Assessment

This case demonstrates that diagnosis of craniofacial FD requires integration of clinical, radiographic, histologic, and molecular findings. While diagnosis is often made by clinical presentation and suggestive radiographic findings, histologic and genetic analyses are recommended in questionable cases. The variable correlation of radiographic, histologic, and molecular diagnosis highlights the clinical heterogeneity of FD and challenges in accurate diagnosis. 13 30

The favorable postoperative outcome supports the appropriateness of surgical intervention for diagnostically uncertain lesions with concerning imaging features. The patient’s complete symptom resolution and stable clinical course at follow-up align with the benign nature of FD. While asymptomatic patients do not require surgical treatment, symptomatic presentations or those with rapid clinical or radiological evolution, as in this case, warrant surgical intervention for both diagnostic certainty and therapeutic benefit. 3 31

## Conclusion

This case underscores the diagnostic challenge of acute symptomatic change in craniofacial FD, particularly in patients with McCune–Albright syndrome. Despite rapid growth and aggressive imaging features, histopathologic and molecular analyses confirmed FD without ABC formation or malignant transformation. These findings demonstrate that concerning radiographic features and fluid accumulation may occur in FD without secondary pathology, emphasizing the need for integrated clinical, imaging, and pathologic evaluation to guide management.
